# Poly[[triaqua­(μ_3_-4-oxidopyridine-2,6-dicarboxyl­ato)europium(III)] monohydrate]

**DOI:** 10.1107/S1600536810048518

**Published:** 2010-11-30

**Authors:** Dong-Yu Lv, Zhu-Qing Gao, Jin-Zhong Gu

**Affiliations:** a Key Laboratory of Nonferrous Metal Chemistry and Resources Utilization of Gansu Province, College of Chemistry and Chemical Engineering, Lanzhou University, Lanzhou, Gansu 730000, People’s Republic of China; b School of Chemistry and Biology Engineering, Taiyuan University of Science and Technology, Taiyuan 030021, People’s Republic of China

## Abstract

In the title coordination polymer, {[Eu(C_7_H_2_NO_5_)(H_2_O)_3_]·H_2_O}_*n*_, the Eu^III^ atom is eight-coordinated by a tridentate 4-oxidopyridine-2,6-dicarboxyl­ate (hpc) trianion, two monodentate hpc anions and three water mol­ecules, forming a distorted bicapped trigonal–prismatic coordination geometry. The hpc ligands bridge adjacent Eu^III^ ions, forming infinite double chains. Adjacent chains are further connected by hpc ligands into sheets. O—H⋯O hydrogen bonds then generate a three-dimensional supra­molecular framework.

## Related literature

For the structures and properties of lanthanide coordination compounds, see: He *et al.* (2010 ▶); Kustaryono *et al.* (2010 ▶); Zhu, Sun *et al.* (2009 ▶); Wong *et al.* (2006 ▶). For the use of multi-carboxyl­ate and heterocyclic acids in coordination chemistry, see: Li *et al.* (2008 ▶); Luo *et al.* (2008 ▶) and for the dicarboxyl­ate ligand H_3_CAM (H_3_CAM is 4-hy­droxy-pyridine-2,6-dicarb­oxy­lic acid), see: Gao *et al.* (2006 ▶, 2008 ▶). For the isotypic structure {[Dy(CAM)(H_2_O)_3_]·H_2_O}_*n*_, see: Gao *et al.* (2006 ▶). For bond lengths and angles in other complexes with eight-coordinate Eu^III^, see: Li *et al.* (2008 ▶); Zhu, Ikarashi *et al.* (2009 ▶) 
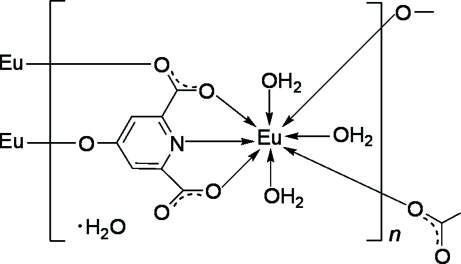


## Experimental

### 

#### Crystal data

[Eu(C_7_H_2_NO_5_)(H_2_O)_3_]·H_2_O
*M* *_r_* = 404.12
Monoclinic, 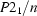

*a* = 10.0041 (15) Å
*b* = 7.5456 (11) Å
*c* = 15.528 (2) Å
β = 104.890 (1)°
*V* = 1132.8 (3) Å^3^
*Z* = 4
Mo *K*α radiation
μ = 5.58 mm^−1^
*T* = 296 K
0.35 × 0.32 × 0.31 mm

#### Data collection

Bruker APEX CCD diffractometer
Absorption correction: multi-scan (*SADABS*; Bruker, 1997 ▶) *T*_min_ = 0.246, *T*_max_ = 0.277
4884 measured reflections
2023 independent reflections
1856 reflections with *I* > 2σ(*I*)
*R*_int_ = 0.074

#### Refinement

*R*[*F* ^2^ > 2σ(*F* ^2^)] = 0.026
*wR*(*F* ^2^) = 0.065
*S* = 1.06
2023 reflections
196 parameters
12 restraints
H atoms treated by a mixture of independent and constrained refinement
Δρ_max_ = 0.82 e Å^−3^
Δρ_min_ = −1.69 e Å^−3^

### 

Data collection: *SMART* (Bruker, 1997 ▶); cell refinement: *SAINT* (Bruker, 1997 ▶); data reduction: *SAINT*; program(s) used to solve structure: *SHELXS97* (Sheldrick, 2008) ▶; program(s) used to refine structure: *SHELXL97* (Sheldrick, 2008) ▶; molecular graphics: *SHELXTL* (Sheldrick, 2008) ▶; software used to prepare material for publication: *SHELXTL* ▶.

## Supplementary Material

Crystal structure: contains datablocks I, global. DOI: 10.1107/S1600536810048518/hb5735sup1.cif

Structure factors: contains datablocks I. DOI: 10.1107/S1600536810048518/hb5735Isup2.hkl

Additional supplementary materials:  crystallographic information; 3D view; checkCIF report

## Figures and Tables

**Table 1:** Selected bond lengths (Å)

Eu1—O5^i^	2.327 (2)
Eu1—O8	2.401 (3)
Eu1—O7	2.416 (3)
Eu1—O1	2.432 (2)
Eu1—O2^ii^	2.433 (2)
Eu1—O3	2.440 (2)
Eu1—O6	2.445 (3)
Eu1—N1	2.498 (3)

Symmetry codes: (i) 
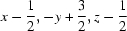
; (ii) 
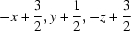
.

**Table 2:** Hydrogen-bond geometry (Å, °)

*D*—H⋯*A*	*D*—H	H⋯*A*	*D*⋯*A*	*D*—H⋯*A*
O6—H1*W*⋯O9^iii^	0.90 (2)	1.85 (3)	2.696 (3)	158 (4)
O6—H2*W*⋯O9^iv^	0.87 (2)	2.15 (3)	2.962 (4)	156 (4)
O7—H4*W*⋯O3^iii^	0.86 (2)	2.09 (3)	2.805 (3)	141 (4)
O8—H5*W*⋯O1^ii^	0.86 (2)	1.84 (2)	2.684 (4)	168 (4)
O8—H6*W*⋯O4^v^	0.87 (2)	1.83 (2)	2.696 (4)	173 (4)
O9—H7*W*⋯O2^vi^	0.86 (2)	2.26 (3)	3.059 (4)	155 (5)
O9—H8*W*⋯O4	0.86 (2)	1.84 (2)	2.692 (4)	172 (6)

Symmetry codes: (ii) 
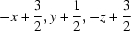
; (iii) 
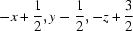
; (iv) 
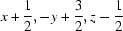
; (v) 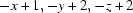; (vi) 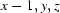.

**Fig. 1.**
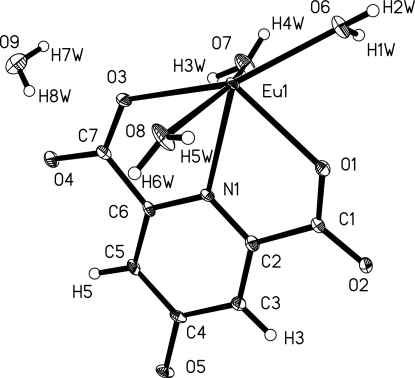
A drawing of the asymmetric unit in the structure of (I), showing displacement ellipsoids drawn at the 30% probability level.

**Fig. 2.**
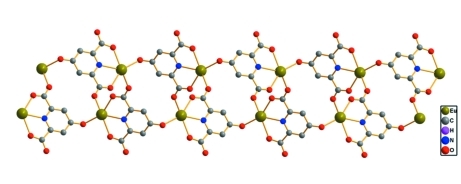
A view along the b axis, showing a one-dimensional double chain of [Eu(CAM)(H2O)3].

**Fig. 3.**
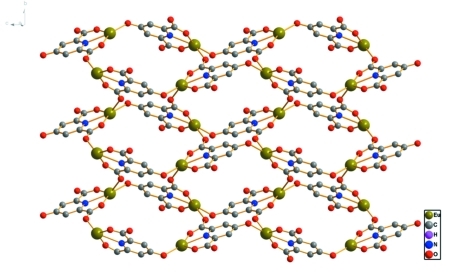
A view along the a axis, showing a two-dimensional sheet of [Eu(CAM)(H2O)3].

## supplementary crystallographic information

### Comment

The design and synthesis of lanthanide coordination polymers have achieved great progress over the past decades(He *et al.*, 2010; Kustaryono *et al.*, 2010). These coordination polymers have shown not only their versatile architectures but also their desirable properties luminescent, magnetic, catalytic, and gas absorption and separation properties (Zhu *et al.*, 2009; Wong *et al.*, 2006). Many multi-carboxylate or heterocylic carboxylic acids are used for this purpose (Li *et al.*, 2008; Luo *et al.*, 2008). In the designed synthesis of the lanthanide coordination polymers, 4-hydroxy-pyridine-2,6-dicarboxylic acid (H_3_CAM) is an excellent pyridine dicarboxylate ligand (Gao *et al.*, 2006; Gao *et al*., 2008),which can afford at most one nitrogen atom and five O coordination sites. In order to extend the investigation in this field, we designed and synthesized one lanthanidecoordination polymer [Eu(CAM)(H_2_O)_3_]*_n_*.nH_2_O, and report its structure here.

The title compound is located on a twofold helical axis of symmetry, which is isomorphous with {[Dy(CAM)(H_2_O)_3_].H_2_O}*_n_* (Gao *et al.*, 2006). As shown in Fig.1, the asymmetrical unit of the cell contains one Eu (III) ion, one CAM liangd, three coordinated water molecules, and one guest water molecule. Eu atom is eight-coordinated with seven oxygen atoms from three individual CAM ligands and three coordinated water molecules and one nitrogen atom from the CAM ligand, forming a distorted bicapped square-prismatic coordination geometry.

Important bond distances and angles are presented in Table 1. The Eu–O bond distances [2.327 (2) to 2.445 (3) Å]are shorter than the Eu–N bond distance [2.498 (3) Å], which are in good with those observed in other Eu (III) complexes (Li *et al.*, 2008; Zhu *et al.*, 2009). The CAM ligands adopt a *µ*_3_-pentadentate coordination mode, as shown in Fig.1. The CAM ligands bridge the adjacent Eu^III^ ions to form infinite double chains (Fig.2). The adjacent chains are further connected by the coordination of the CAMligands and Eu^III^ ions to form two-dimensional sheet (Fig.3), which are further extended into three-dimensional supramolecular frameworks through H-bond interactions (Table 4).

### Experimental

To a solution of europium nitrate hexahydrate (0.134 g, 0.3 mmol) in water (5 ml) was added an aqueous solution (5 ml) of the ligand (0.060 g, 0.3 mmol) and a drop of triethylamine. The reactants were sealed in a 25-ml Teflon-lined, stainless-steel Parr bomb. The bomb was heated at 433 K for 3 days. The cool solution yielded colourless blocks in *ca* 60% yield. Anal. Calcd for C_7_H_10_EuNO_9_: C, 20.80; H, 2.49; N, 3.47. Found: C, 20.51; H, 2.77; N, 3.12.

### Refinement

The coordinated water H atoms were located in a different Fourier map and refined with distance constraints of O–H = 0.83 (3) Å. The free water H atoms attached to oxygen atoms were placed at calculated positions and refined with the riding model, considering the position of oxygen atoms and the quantity of H atoms. The carbon-bound H atoms were placed in geometrically idealized positions, with C–H = 0.93 Å, and constrained to ride on their respective parent atoms, with *U*iso(H) = 1.2 *U*eq(C).

### Crystal data

**Table:**

[Eu(C_7_H_2_NO_5_)(H_2_O)_3_]·H_2_O	*F*(000) = 776
*M**_r_* = 404.12	*D*_x_ = 2.370 Mg m^−^^3^
Monoclinic, *P*2_1_/*n*	Mo *K*α radiation, λ = 0.71073 Å
Hall symbol: -P 2yn	Cell parameters from 3455 reflections
*a* = 10.0041 (15) Å	θ = 2.8–28.3°
*b* = 7.5456 (11) Å	µ = 5.58 mm^−^^1^
*c* = 15.528 (2) Å	*T* = 296 K
β = 104.890 (1)°	Block, colorless
*V* = 1132.8 (3) Å^3^	0.35 × 0.32 × 0.31 mm
*Z* = 4	

### Data collection

**Table:**

Bruker APEXII CCD diffractometer	2023 independent reflections
Radiation source: fine-focus sealed tube	1856 reflections with *I* > 2σ(*I*)
graphite	*R*_int_ = 0.074
φ and ω scans	θ_max_ = 25.5°, θ_min_ = 2.8°
Absorption correction: multi-scan (*SADABS*; Bruker, 1997)	*h* = −11→12
*T*_min_ = 0.246, *T*_max_ = 0.277	*k* = −9→7
4884 measured reflections	*l* = −14→18

### Refinement

**Table:**

Refinement on *F*^2^	Secondary atom site location: difference Fourier map
Least-squares matrix: full	Hydrogen site location: inferred from neighbouring sites
*R*[*F*^2^ > 2σ(*F*^2^)] = 0.026	H atoms treated by a mixture of independent and constrained refinement
*wR*(*F*^2^) = 0.065	*w* = 1/[σ^2^(*F*_o_^2^) + 0.5803*P*] where *P* = (*F*_o_^2^ + 2*F*_c_^2^)/3
*S* = 1.06	(Δ/σ)_max_ = 0.001
2023 reflections	Δρ_max_ = 0.82 e Å^−^^3^
196 parameters	Δρ_min_ = −1.69 e Å^−^^3^
12 restraints	Extinction correction: *SHELXL97* (Sheldrick, 2008), Fc^*^=kFc[1+0.001xFc^2^λ^3^/sin(2θ)]^-1/4^
Primary atom site location: structure-invariant direct methods	Extinction coefficient: 0.0273 (8)

### Special details

**Table:**

Geometry. All e.s.d.'s (except the e.s.d. in the dihedral angle between two l.s. planes) are estimated using the full covariance matrix. The cell e.s.d.'s are taken into account individually in the estimation of e.s.d.'s in distances, angles and torsion angles; correlations between e.s.d.'s in cell parameters are only used when they are defined by crystal symmetry. An approximate (isotropic) treatment of cell e.s.d.'s is used for estimating e.s.d.'s involving l.s. planes.
Refinement. Refinement of *F*^2^ against ALL reflections. The weighted *R*-factor *wR* and goodness of fit *S* are based on *F*^2^, conventional *R*-factors *R* are based on *F*, with *F* set to zero for negative *F*^2^. The threshold expression of *F*^2^ > σ(*F*^2^) is used only for calculating *R*-factors(gt) *etc*. and is not relevant to the choice of reflections for refinement. *R*-factors based on *F*^2^ are statistically about twice as large as those based on *F*, and *R*- factors based on ALL data will be even larger.

### Fractional atomic coordinates and isotropic or equivalent isotropic displacement parameters (Å^2^)

**Table:**

	*x*	*y*	*z*	*U*_iso_*/*U*_eq_	
Eu1	0.500412 (13)	0.82415 (2)	0.746168 (10)	0.01204 (13)	
C1	0.7849 (3)	0.5972 (4)	0.8389 (2)	0.0179 (7)	
C2	0.7266 (3)	0.6188 (4)	0.9168 (2)	0.0159 (7)	
C3	0.7942 (3)	0.5648 (4)	1.0015 (2)	0.0168 (7)	
H3	0.8789	0.5069	1.0112	0.020*	
H5	0.5632	0.7067	1.0980	0.020*	
C4	0.7362 (3)	0.5967 (4)	1.0731 (2)	0.0153 (7)	
C5	0.6069 (3)	0.6829 (4)	1.0528 (2)	0.0164 (8)	
C6	0.5456 (3)	0.7327 (4)	0.9664 (2)	0.0142 (7)	
C7	0.4087 (3)	0.8280 (4)	0.9380 (2)	0.0163 (8)	
H1W	0.492 (5)	0.543 (3)	0.609 (3)	0.073 (17)*	
H2W	0.536 (5)	0.692 (5)	0.567 (3)	0.060 (17)*	
H3W	0.327 (5)	0.552 (7)	0.785 (2)	0.08 (2)*	
H4W	0.315 (4)	0.511 (6)	0.6919 (18)	0.071 (17)*	
H5W	0.660 (4)	1.125 (6)	0.802 (2)	0.069 (17)*	
H6W	0.623 (4)	1.093 (6)	0.8862 (12)	0.047 (13)*	
H7W	0.034 (5)	0.735 (6)	0.899 (4)	0.11 (2)*	
H8W	0.155 (2)	0.835 (6)	0.949 (4)	0.078 (19)*	
N1	0.6033 (3)	0.7025 (3)	0.89845 (18)	0.0151 (6)	
O1	0.7227 (3)	0.6764 (3)	0.76842 (16)	0.0270 (7)	
O2	0.8922 (2)	0.5058 (3)	0.84687 (15)	0.0234 (6)	
O3	0.3689 (2)	0.8704 (3)	0.85661 (15)	0.0211 (5)	
O4	0.3426 (2)	0.8572 (3)	0.99465 (16)	0.0245 (6)	
O5	0.8011 (2)	0.5511 (3)	1.15508 (14)	0.0202 (5)	
O6	0.5004 (3)	0.6614 (3)	0.61007 (18)	0.0275 (6)	
O7	0.3631 (3)	0.5587 (4)	0.73985 (19)	0.0323 (7)	
O8	0.6026 (3)	1.0809 (4)	0.82870 (18)	0.0319 (7)	
O9	0.0664 (3)	0.8216 (3)	0.9349 (2)	0.0367 (8)	

### Atomic displacement parameters (Å^2^)

**Table:**

	*U*^11^	*U*^22^	*U*^33^	*U*^12^	*U*^13^	*U*^23^
Eu1	0.01379 (17)	0.01728 (17)	0.00543 (16)	0.00006 (5)	0.00313 (11)	0.00080 (5)
C1	0.0194 (16)	0.0240 (17)	0.0107 (17)	0.0022 (14)	0.0047 (14)	−0.0012 (14)
C2	0.0192 (16)	0.0166 (16)	0.0130 (17)	0.0000 (13)	0.0060 (14)	0.0001 (14)
C3	0.0189 (16)	0.0193 (16)	0.0119 (16)	0.0030 (13)	0.0031 (14)	0.0014 (13)
C4	0.0194 (15)	0.0171 (16)	0.0079 (16)	−0.0042 (13)	0.0010 (13)	0.0026 (13)
C5	0.0186 (17)	0.0243 (18)	0.0094 (17)	−0.0032 (12)	0.0092 (14)	−0.0015 (13)
C6	0.0195 (17)	0.0153 (15)	0.0094 (16)	−0.0007 (13)	0.0068 (14)	−0.0007 (13)
C7	0.0180 (17)	0.0193 (17)	0.0109 (18)	−0.0021 (13)	0.0028 (15)	−0.0021 (13)
N1	0.0175 (14)	0.0206 (13)	0.0071 (14)	0.0027 (11)	0.0033 (12)	0.0021 (11)
O1	0.0276 (14)	0.0468 (17)	0.0090 (13)	0.0166 (11)	0.0092 (11)	0.0085 (11)
O2	0.0241 (13)	0.0330 (14)	0.0128 (12)	0.0135 (11)	0.0043 (10)	−0.0014 (11)
O3	0.0201 (11)	0.0338 (13)	0.0094 (12)	0.0077 (11)	0.0041 (10)	0.0031 (11)
O4	0.0236 (12)	0.0419 (15)	0.0110 (13)	0.0061 (11)	0.0097 (11)	−0.0009 (11)
O5	0.0206 (11)	0.0305 (13)	0.0087 (12)	−0.0030 (10)	0.0021 (10)	0.0042 (10)
O6	0.0423 (17)	0.0266 (15)	0.0169 (15)	−0.0036 (12)	0.0135 (14)	−0.0033 (11)
O7	0.0413 (16)	0.0376 (16)	0.0212 (15)	−0.0181 (13)	0.0141 (14)	−0.0058 (13)
O8	0.0451 (16)	0.0393 (16)	0.0164 (14)	−0.0194 (14)	0.0172 (13)	−0.0085 (12)
O9	0.0278 (16)	0.0311 (16)	0.049 (2)	0.0034 (12)	0.0062 (15)	0.0025 (13)

### Geometric parameters (Å, °)

**Table:**

Eu1—O5^i^	2.327 (2)	C5—C6	1.377 (4)
Eu1—O8	2.401 (3)	C5—H5	0.9344
Eu1—O7	2.416 (3)	C6—N1	1.345 (4)
Eu1—O1	2.432 (2)	C6—C7	1.509 (4)
Eu1—O2^ii^	2.433 (2)	C7—O4	1.249 (4)
Eu1—O3	2.440 (2)	C7—O3	1.264 (4)
Eu1—O6	2.445 (3)	O2—Eu1^iii^	2.433 (2)
Eu1—N1	2.498 (3)	O5—Eu1^iv^	2.327 (2)
C1—O2	1.255 (4)	O6—H1W	0.895 (19)
C1—O1	1.262 (4)	O6—H2W	0.867 (19)
C1—C2	1.481 (5)	O7—H3W	0.871 (19)
C2—N1	1.350 (4)	O7—H4W	0.856 (19)
C2—C3	1.376 (4)	O8—H5W	0.855 (19)
C3—C4	1.402 (5)	O8—H6W	0.868 (18)
C3—H3	0.9300	O9—H7W	0.861 (19)
C4—O5	1.317 (3)	O9—H8W	0.861 (19)
C4—C5	1.410 (4)		
			
O5^i^—Eu1—O8	100.29 (9)	O2—C1—C2	119.1 (3)
O5^i^—Eu1—O7	85.49 (9)	O1—C1—C2	116.5 (3)
O8—Eu1—O7	148.21 (10)	N1—C2—C3	122.6 (3)
O5^i^—Eu1—O1	151.83 (8)	N1—C2—C1	114.1 (3)
O8—Eu1—O1	92.61 (9)	C3—C2—C1	123.2 (3)
O7—Eu1—O1	96.63 (9)	C2—C3—C4	120.4 (3)
O5^i^—Eu1—O2^ii^	81.44 (8)	C2—C3—H3	119.8
O8—Eu1—O2^ii^	70.73 (9)	C4—C3—H3	119.8
O7—Eu1—O2^ii^	140.91 (9)	O5—C4—C3	121.4 (3)
O1—Eu1—O2^ii^	79.36 (8)	O5—C4—C5	122.2 (3)
O5^i^—Eu1—O3	80.61 (8)	C3—C4—C5	116.4 (3)
O8—Eu1—O3	75.04 (9)	C6—C5—C4	119.8 (3)
O7—Eu1—O3	75.15 (9)	C6—C5—H5	120.2
O1—Eu1—O3	127.18 (8)	C4—C5—H5	120.0
O2^ii^—Eu1—O3	137.46 (8)	N1—C6—C5	123.0 (3)
O5^i^—Eu1—O6	82.43 (9)	N1—C6—C7	113.1 (3)
O8—Eu1—O6	140.55 (10)	C5—C6—C7	123.9 (3)
O7—Eu1—O6	71.01 (10)	O4—C7—O3	124.9 (3)
O1—Eu1—O6	71.90 (9)	O4—C7—C6	118.9 (3)
O2^ii^—Eu1—O6	70.83 (9)	O3—C7—C6	116.2 (3)
O3—Eu1—O6	143.08 (9)	C6—N1—C2	117.8 (3)
O5^i^—Eu1—N1	143.53 (9)	C6—N1—Eu1	121.6 (2)
O8—Eu1—N1	77.05 (9)	C2—N1—Eu1	120.3 (2)
O7—Eu1—N1	79.95 (9)	C1—O1—Eu1	124.7 (2)
O1—Eu1—N1	63.77 (9)	C1—O2—Eu1^iii^	138.4 (2)
O2^ii^—Eu1—N1	129.18 (9)	C7—O3—Eu1	125.3 (2)
O3—Eu1—N1	63.42 (8)	C4—O5—Eu1^iv^	127.88 (19)
O6—Eu1—N1	122.83 (9)	Eu1—O6—H1W	119 (3)
O5^i^—Eu1—H5W	101.3 (10)	Eu1—O6—H2W	129 (3)
O8—Eu1—H5W	17.0 (6)	H1W—O6—H2W	108 (3)
O7—Eu1—H5W	164.4 (6)	Eu1—O7—H3W	111 (3)
O1—Eu1—H5W	84.1 (10)	Eu1—O7—H4W	125 (3)
O2^ii^—Eu1—H5W	54.6 (6)	H3W—O7—H4W	115 (3)
O3—Eu1—H5W	91.9 (6)	Eu1—O8—H5W	108 (3)
O6—Eu1—H5W	123.6 (6)	Eu1—O8—H6W	126 (3)
N1—Eu1—H5W	86.4 (8)	H5W—O8—H6W	116 (3)
O2—C1—O1	124.4 (3)	H7W—O9—H8W	116 (3)
			
O2—C1—C2—N1	−173.0 (3)	O6—Eu1—N1—C6	−143.3 (2)
O1—C1—C2—N1	8.2 (4)	O5^i^—Eu1—N1—C2	170.8 (2)
O2—C1—C2—C3	9.4 (5)	O8—Eu1—N1—C2	−99.4 (2)
O1—C1—C2—C3	−169.3 (3)	O7—Eu1—N1—C2	102.7 (2)
N1—C2—C3—C4	−0.4 (5)	O1—Eu1—N1—C2	0.1 (2)
C1—C2—C3—C4	177.0 (3)	O2^ii^—Eu1—N1—C2	−48.0 (3)
C2—C3—C4—O5	−177.9 (3)	O3—Eu1—N1—C2	−178.9 (3)
C2—C3—C4—C5	0.9 (5)	O6—Eu1—N1—C2	43.2 (3)
O5—C4—C5—C6	178.0 (3)	O2—C1—O1—Eu1	172.3 (2)
C3—C4—C5—C6	−0.8 (4)	C2—C1—O1—Eu1	−9.0 (4)
C4—C5—C6—N1	0.2 (5)	O5^i^—Eu1—O1—C1	−163.2 (2)
C4—C5—C6—C7	−179.0 (3)	O8—Eu1—O1—C1	79.2 (3)
N1—C6—C7—O4	177.2 (3)	O7—Eu1—O1—C1	−70.3 (3)
C5—C6—C7—O4	−3.5 (5)	O2^ii^—Eu1—O1—C1	149.0 (3)
N1—C6—C7—O3	−1.8 (4)	O3—Eu1—O1—C1	6.0 (3)
C5—C6—C7—O3	177.4 (3)	O6—Eu1—O1—C1	−137.8 (3)
C5—C6—N1—C2	0.3 (5)	N1—Eu1—O1—C1	5.0 (3)
C7—C6—N1—C2	179.6 (3)	O1—C1—O2—Eu1^iii^	−29.7 (5)
C5—C6—N1—Eu1	−173.3 (2)	C2—C1—O2—Eu1^iii^	151.6 (2)
C7—C6—N1—Eu1	6.0 (4)	O4—C7—O3—Eu1	177.6 (2)
C3—C2—N1—C6	−0.2 (5)	C6—C7—O3—Eu1	−3.4 (4)
C1—C2—N1—C6	−177.8 (3)	O5^i^—Eu1—O3—C7	178.4 (3)
C3—C2—N1—Eu1	173.5 (2)	O8—Eu1—O3—C7	−78.2 (3)
C1—C2—N1—Eu1	−4.1 (4)	O7—Eu1—O3—C7	90.6 (3)
O5^i^—Eu1—N1—C6	−15.8 (3)	O1—Eu1—O3—C7	3.6 (3)
O8—Eu1—N1—C6	74.1 (2)	O2^ii^—Eu1—O3—C7	−115.4 (2)
O7—Eu1—N1—C6	−83.8 (2)	O6—Eu1—O3—C7	114.7 (2)
O1—Eu1—N1—C6	173.6 (3)	N1—Eu1—O3—C7	4.6 (2)
O2^ii^—Eu1—N1—C6	125.5 (2)	C3—C4—O5—Eu1^iv^	69.8 (4)
O3—Eu1—N1—C6	−5.5 (2)	C5—C4—O5—Eu1^iv^	−108.9 (3)

Symmetry codes: (i) *x*−1/2, −*y*+3/2, *z*−1/2; (ii) −*x*+3/2, *y*+1/2, −*z*+3/2; (iii) −*x*+3/2, *y*−1/2, −*z*+3/2; (iv) *x*+1/2, −*y*+3/2, *z*+1/2.

### Hydrogen-bond geometry (Å, °)

**Table:**

*D*—H···*A*	*D*—H	H···*A*	*D*···*A*	*D*—H···*A*
O6—H1W···O9^v^	0.90 (2)	1.85 (3)	2.696 (3)	158 (4)
O6—H2W···O9^vi^	0.87 (2)	2.15 (3)	2.962 (4)	156 (4)
O7—H4W···O3^v^	0.86 (2)	2.09 (3)	2.805 (3)	141 (4)
O8—H5W···O1^ii^	0.86 (2)	1.84 (2)	2.684 (4)	168 (4)
O8—H6W···O4^vii^	0.87 (2)	1.83 (2)	2.696 (4)	173 (4)
O9—H7W···O2^viii^	0.86 (2)	2.26 (3)	3.059 (4)	155 (5)
O9—H8W···O4	0.86 (2)	1.84 (2)	2.692 (4)	172 (6)

Symmetry codes: (v) −*x*+1/2, *y*−1/2, −*z*+3/2; (vi) *x*+1/2, −*y*+3/2, *z*−1/2; (ii) −*x*+3/2, *y*+1/2, −*z*+3/2; (vii) −*x*+1, −*y*+2, −*z*+2; (viii) *x*−1, *y*, *z*.
